# MicroRNA‐binding site polymorphisms and risk of colorectal cancer: A systematic review and meta‐analysis

**DOI:** 10.1002/cam4.2600

**Published:** 2019-10-21

**Authors:** Morteza Gholami, Bagher Larijani, Farshad Sharifi, Shirin Hasani‐Ranjbar, Reza Taslimi, Milad Bastami, Rasha Atlasi, Mahsa M. Amoli

**Affiliations:** ^1^ Obesity and Eating Habits Research Center Endocrinology and Metabolism Clinical Sciences Institute Tehran University of Medical Sciences Tehran Iran; ^2^ Endocrinology and Metabolism Research Center Endocrinology and Metabolism Clinical Sciences Institute Tehran University of Medical Sciences Tehran Iran; ^3^ Elderly Health Research Center Endocrinology and Metabolism Population Sciences Institute Tehran University of Medical Sciences Tehran Iran; ^4^ Department of Gastroenterology Imam Khomeini Hospital Tehran University of Medical Sciences Tehran Iran; ^5^ Department of Medical Genetics Faculty of Medicine Tabriz University of Medical Sciences Tabriz Iran; ^6^ Evidence Based Practice Research Center Endocrinology and Metabolism Clinical Sciences Institute Tehran University of Medical Sciences Tehran Iran; ^7^ Metabolic Disorders Research Center Endocrinology and Metabolism Molecular‐Cellular Sciences Institute Tehran University of Medical Sciences Tehran Iran

**Keywords:** colorectal cancer, meta‐analysis, microRNAs, polymorphism

## Abstract

Genetic variations in miRNAs binding site might participate in cancer risk. This study aimed to systematically review the association between miRNA‐binding site polymorphisms and colorectal cancer (CRC). Electronic literature search was carried out on PubMed, Web of Science (WOS), Scopus, and Embase. All types of observational studies till 30 November 2018 were included. Overall 85 studies (21 SNPs) from two systematic searches were included analysis. The results showed that in the Middle East population, the minor allele of rs731236 was associated with decreased risk of CRC (heterozygote model: 0.76 [0.61‐0.95]). The minor allele of rs3025039 was related to increased risk of CRC in East Asian population (allelic model: 1.25 [1.01‐1.54]). Results for rs3212986 were significant in overall and subgroup analysis (*P* < .05). For rs1801157 in subgroup analysis the association was significant in Asian populations (including allelic model: 2.28 [1.11‐4.69]). For rs712, subgroup analysis revealed a significant (allelic model: 1.41 [1.23‐1.61]) and borderline (allelic model: 0.92 [0.84‐1.00]) association in Chinese and Czech populations, respectively. The minor allele of rs17281995 increased risk of CRC in different genetic models (*P* < .05). Finally, rs5275, rs4648298, and rs61764370 did not show significant associations. In conclusion, minor allele of rs3025039, rs3212986, and rs712 polymorphisms increases the risk of CRC in the East Asian population, and heterozygote model of rs731236 polymorphism shows protective effect in the Middle East population. In Europeans, the minor allele of rs17281995 may increase the risk of CRC, while rs712 may have a protective effect. Further analysis based on population stratifications should be considered in future studies.

## Graphical abstract

MicroRNA‐binding site polymorphisms in 3'UTR of target genes could play critical roles on cancer genes regulation by affecting miRNA:mRNA interactions, but no comprehensive study has been considered on association between cancers and the mentioned polymorphisms. In this study, the authors carried out two comprehensive systematic searches and a meta‐analysis on 21 included polymorphisms on colorectal cancer (CRC). The results have shown that these polymorphisms can serve as genetic biomarkers of CRC, and their roles were exclusively related to population stratifications. It is strongly recommended that further studies should be performed to display the effect of population stratifications.

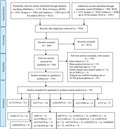

## 1 INTRODUCTION

Colorectal cancer is one of the most serious illnesses in both sexes. It has been recognized as the second and third common cancers in females and males, respectively.^1^, ^2^, ^3^ Incidence and mortality of colorectal cancer (CRC)was about 6.1% of new cancer cases and was around 9.2% of cancer death based on Global Cancer Statistics 2018.^4^ Its incidence is three times higher in developed countries than developing counters.^4^ CRC imposes enormous global burden which could be related to aging and population growth, socioeconomic status, diet, life styles, and habits including smoking, western diet, and physical activity.^5^, ^6^, ^7^ Early diagnosis of CRC leads to lesser treatment cost besides better survival and prognosis.^8^ Early prognosis or diagnosis of CRC is also important in cancer survival. Nine of 10 people with CRC would have more than 5 years of survival, if the diagnosis is performed at the stage one while diagnosis in the last stage leads to merely 1 year of survival. For this purpose, finding novel biomarkers for noninvasive early diagnosis of CRC will be crucial in disease treatment.

Some risk factors of CRC including diet and smoking could be modified in contrast to genetic factors.^9^, ^10^, ^11^ MicroRNAs (miRNAs) are important genetic factors which are regulating around 60% of human protein‐coding genes.^12^ It is believed that miRNAs play an important role in the pathogenesis of CRC.^13^ miRNA polymorphisms might participate in cancer prognosis through their effect on miRNA gene transcription, processing, expression, and target selection.^14^, ^15^, ^16^ A meta‐analysis in 2016 has been implemented on the association between miR‐27a rs895819 in the loop of pre‐miRNA and shows that this SNP may be a risk factor for CRC (for instance in allelic model OR = 1.21 [1.11‐1.31]).^13^ A systematic review and meta‐analysis has been published in 2014 based on the role of two polymorphisms in miR‐146a and in miR‐196a2 on the susceptibility towards CRC. The results revealed that miR‐196a2 polymorphism rs11614913 is associated with the risk of CRC.^17^ Another review paper in 2015 described the association of miRNA variants (in miR‐146a, hsa‐miR‐149, and hsa‐miR‐196a2) and CRC and showed that rs2910164 (1.24 [1.03‐1.49]) and rs2292832 (1.18 [1.08‐1.38]) may increase the risk of CRC, and rs11614913 and rs3746444 (0.57 [0.34‐0.95]) may decrease the risk of CRC.^18^ In 2017, a review article was published on the risk of CRC and polymorphisms in microRNA gene. Based on these results let‐7, miR‐149, miR‐603, miR‐34b/c, and miR‐146a gene SNPs were associated with CRC.^19^

Polymorphisms in miRNA‐binding sites may also alter the risk and survival of a variety of human complex diseases including CRC.^20^, ^21^, ^22^ miRNA‐binding sites are conserved through evolution and contain lesser polymorphisms.^23^ Polymorphisms in these sites can affect miRNA:mRNA interactions and target mRNA expression.^24^, ^25^ In one study, the association between let‐7 miRNA‐binding site polymorphisms and CRC outcome has been described, based on one miRNA, one database (PubMed), and also CRC risk was not investigated.^26^ miRNAs’ target site polymorphisms may potentially play a role in the interaction between miRNAs and their target mRNA, which is dependent on the effect of polymorphism on miRNA:mRNA interactions. There was also a meta‐analysis on 3'UTR polymorphisms and the risk of cancers,^27^ but the results were only for two polymorphisms and were not specific for CRC or miRNA‐binding sites. To the best of our knowledge, there is no previous systematic review on the association between miRNA‐binding site polymorphisms and CRC. Therefore, the lack of a comprehensive systematic review focusing on miRNA‐binding site polymorphisms and CRC is obvious.

Because of importance and economic burden of CRC, and regarding the significant role of miRNA‐binding site polymorphisms on CRC according to the previous studies besides lack of a systematic review on this subject, the necessity of such study on association between miRNA‐binding site polymorphisms and CRC, as prognostic markers, is quite clear. For this purpose, the main objective of the current systematic review was to explore and reveal the association of 3'UTR and miRNA‐binding site polymorphisms with the risk of CRC. The secondary specific objective was to determine the effect of ethnicity on these associations.

## 2 METHODS AND ANALYSIS

**Figure 1:**
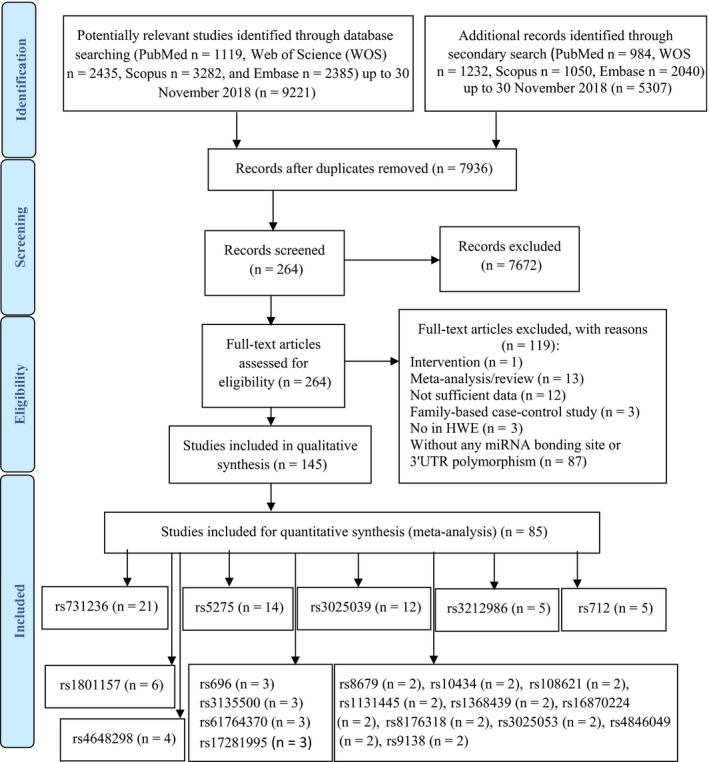
Flow diagram for systematic review

The methods of this study have been developed according to the PRISMA‐P 2015 checklist.^28^ PRISMA 2009 flow diagram,^29^ used to display the flow of document number through the different phases of the study (Figure 1). The protocol of this systematic review is registered in International Prospective Register for Systematic Reviews (PROSPERO) on January 11, 2018 (Registration ID = CRD42018084094).

### 2.1 Eligible studies and participants

This study imposed a restriction on the study design. Observational studies (case‐control, cohort, and cross‐sectional), describing the association between miRNA‐binding site polymorphisms and CRC, were eligible for inclusion. Primary documents will be screened according to the PECO criteria (Participants, Exposure, Comparisons, and Outcomes) and objectives of this study. Studies with deviation from Hardy‐Weinberg equilibrium^30^ (HWE) and with the lack of required primary data or data for estimating genotype numbers were excluded. This study also applied a restriction on publication date. Only documents published from January 1, 1992 to November 30, 2018 were searched. This restriction was based on two reasons; first: miRNA discovery date, and second: most recent publications were relevant to our study subject. There was no restriction about the language of documents related to the topic of this study. Non‐English languages articles were translated by free language translation services or by a translator. There was also no limitation on age, gender, ethnicity, and method of genotyping. The study did not impose a restriction on colorectal cancer stages (I, II, III, and IV). Colorectal polyps and family‐based case‐control studies were not considered for inclusion.

### 2.2 MicroRNAs binding site polymorphism

Polymorphisms in miRNA‐binding sites have been reported to be associated with cancers.^31^, ^32^ These SNPs are conserved through evolution.^23^ These sites act as diagnostic and prognostic biomarkers associated with cancer risk and outcome.^33^ Their association with susceptibility, outcome, treatment, prognosis, and progression of CRC has also been reported.^20^, ^34^, ^35^, ^36^ In this systematic review, studies that evaluated the relationship between miRNA‐binding site polymorphisms and CRC were included and the primary outcome of this review was finding association between miRNA‐binding site polymorphisms and CRC susceptibility. Moreover, subgroup analysis for ethnicity was carried out on association of CRC risk with microRNA‐binding site polymorphisms.

### 2.3 Search methods for studies identification

In order to identify the relevant papers on miRNA‐binding site polymorphisms and colorectal cancer, online systematic search (electronic searches) of literature was performed in PubMed, Embase, Scopus, and Web of Science. We developed PubMed search syntax, as the main database, this syntax was adapted to other database. PubMed search syntax was performed by combined medical subject headings (MeSH), Emtree terms, keywords of related papers, also free text words. Key search terms were “colorectal neoplasms,” “miRNA,” “Polymorphism, Single Nucleotide,” and their equivalents (Table S1). To identify more results, we also manually checked references from included primary articles and relevant reviews, conference papers, gray literature, as well as contact with corresponding authors for missing data.

### 2.4 Data collection

#### 2.4.1 Screening for eligible studies

Screening and eligibility checking was performed in three following steps. First, duplicate documents were removed. Second, for screening, two reviewers independently scrutinize remaining documents by checking title and/or abstract. Third, full texts' eligibility was independently scrutinized by two reviewers. Any disagreements between two reviewers were resolved by consensus strategy and third‐person strategy.

#### 2.4.2 Data extraction and management

A data extraction form was created and then piloted by two reviewers. This form included the following data: the name of first author, country of study, year of publication, study design, age, gender, ethnicity, names of 3'UTR or binding site SNPs, genotyping methods, minor allele frequency (MAF), HWE, sample size, matching criteria (such as age and sex), source of controls (HB, hospital base or PB, population base), odds ratio (OR), confidence interval (95% CIs), and other related raw data. In the next step, two reviewers independently extracted data based on the extraction form. Disagreements were resolved by strategies listed above.

### 2.5 Analysis

#### 2.5.1 Meta‐analysis

Meta‐analysis was performed by using R (3.5.2). Odds ratio and 95% CI were used to investigate the associations between each polymorphism in miRNA‐binding site and CRC. The meta‐analysis was performed based on different genetic models (allelic model (A vs a), homozygous model (AA vs aa), heterozygote model (Aa vs aa), AA vs Aa model, dominant model (AA + Aa vs aa), recessive model (AA vs Aa + aa), and overdominant model (Aa vs AA + aa)). All included studies were at the risk of various types of heterogeneity. For exploring possible sources of heterogeneity, included studies were divided according to the type of polymorphisms. For each polymorphism, if sufficient studies were included, subgroup analysis (based on ethnicity) was applied. Odds ratios were estimated by fixed effects model (FEM) or random effects model (REM), according to the heterogeneity level. Level of heterogeneity between primary studies was obtained by the Cochran's Q test (*P* < .05 is statistically significant) and the *I* ^2^ statistic in forest plots. We used the following guide to interpret the amount of heterogeneity: *I* ^2^ < 25% = low heterogeneity; 25 ≥ *I* ^2^ < 50% = moderate heterogeneity; 50 ≥ *I* ^2^ < 75% = sever heterogeneity; 75% ≥ *I* ^2^ = highly sever heterogeneity.

#### 2.5.2 Reporting biases and sensitivity analysis

We used Begg's test and Egger's regression method to assess the potential publication bias in primary studies. Main results were depicted by funnel plots (for visual assessment). Sensitivity analysis was performed by the leave‐one‐out method.

## 3 RESULTS

In the systematic search, at the first stage we found 9221 documents, with 222 polymorphisms in 3′UTR and miRNA‐binding site of genes that were studied for the risk of CRC. Among them we included main polymorphisms in second search for meta‐analysis (these polymorphisms were selected because the meta‐analysis for all included polymorphisms was not possible, also in order to decrease the false positive prediction of miRNA‐binding sites polymorphisms, only polymorphisms that were mentioned in two studies or more were included, one of these studies should report polymorphism in miRNA‐binding site). Twenty‐five polymorphisms were included (rs10082466, rs10434, rs8176318, rs17281995, rs3212986, rs1368439, rs1131445, rs5275, rs61764370, rs712, rs108621, rs696, rs3135500, rs8679, rs16870224, rs731236, rs3025039, rs3025040, rs3025053, rs4648298, rs1801157, rs3742330, rs4846049, rs854551, and rs9138). Second search strategy applied for these polymorphisms, which contained 5170 documents. Finally, we included 54 studies on the role of 3′UTR polymorphisms and 52 studies on the role of miRNA‐binding site polymorphisms and risk of CRC for all the selected polymorphisms (Tables 1 and 2). Finally, 21 polymorphisms with two or more than two included studies were eligible for final analysis (these studies are shown in detail in Tables 3 and 4). For rs17281995 polymorphism, the pooled analysis based on three included articles showed significant increased risk of CRC in different genetic models, including homozygote model 2.29 (1.25‐4.19). Seven of 21 included polymorphisms in our meta‐analysis were polymorphisms with more than four included articles (rs731236, rs3025039, rs3212986, rs712, rs5275, rs4648298, and rs1801157). The basic characteristics of studies included in the meta‐analysis are shown following (Table 4).

**Table 1:** miRNA‐binding sites polymorphisms and colorectal cancer risk (included from first search strategy)

References	Study design	rsID (target miRNA)
37	Case‐control	rs10082466 (miR‐27a)
38	Case‐control	rs11466537 (miR‐1193)
39	Case‐control	rs12904 (miR‐200 family: miR‐200c, miR‐429, and miR‐200b)
40	Case‐control	rs12915554 (miR‐185‐3p)
41	Case‐control	rs141178472 (miR‐520a)
42	Case‐control	rs16917496 (miR‐502)
43	Case‐control	rs1710 (miRNA‐binding site polymorphism^a^)
44	Case‐control	rs2015 (miR‐376a‐5p)
45	Case‐control	rs2737 (miR‐379)
46	Case‐control	rs3135500 (miR‐158, miR‐215, miR‐98, miR‐573)
47	Case‐control	rs11169571 (miR‐1283, miR‐520d‐5p)
48	Case‐control	rs34149860 (miR‐29b)
49	Case‐control	rs4648298 (miR‐21, miR590)
50	Case‐control	rs3814058 (miR‐129‐5p)
51	Case‐control	rs4245739 (miR‐191)
52	Case‐control	rs4804800 (miR‐622, miR‐1238)
53	Case‐control	rs4939827 (miR‐375)
54	Case‐control	rs5275 (miR‐542‐3p)
55	Case‐control	rs61764370 (let‐7)
56	Case‐control	rs61764370 (let‐7)
57	Case‐control	rs696 (miR449a)
58	Case‐control	rs696 (miR‐449a, miR‐34b)
36	Case‐control	rs712 (let‐7)
59	Case‐control	rs712 (miR‐200b, miR‐429, miR‐200c, miR‐193b)
60	Case‐control	rs8679 (miR‐145)
61	Case‐control	rs12997 (miR‐330‐3p), rs1043784 (miR‐584), rs10038999 (miR‐629), rs1129976 (miR‐150)
62	Case‐control	rs712 (let‐7), rs61764370 (let‐7)
63	Case‐control	rs17468, rs2317676 (miRNA‐binding site polymorphisms)
64	Case‐control	rs3135500, rs1368439 (miRNA ‐binding site polymorphisms)
65	Case‐control	rs13347 (miR‐509‐3p), rs10836347, rs11821102 (miRNA‐binding site polymorphisms)
66	Case‐control	rs5186 (miR‐155), rs710100 (miR‐155), rs411103 (miR‐27b)
67	Case‐control	rs847 (miR‐98, let‐7i/f/g), rs848 (miR‐558, miR‐621, let‐7i), rs1295685 (miR‐621)
68	Case‐control	rs7930 (miR‐4273‐5p), rs8117825 (miR‐3126‐5p, miR‐337‐3p), rs16853287 (miR‐128‐3p, miR‐140‐3p)
69	Case‐control	rs1590 (miR‐532‐5p, miR‐768‐3p), rs1434536, rs17023107 (miRNA‐binding site polymorphisms)
70	Case‐control	rs4143815 (miR‐570), rs1059293, rs27194, rs43216 (miRNA‐binding site polymorphisms)
71	Case‐control	rs1062044 (miR‐423‐5p), rs17477864 (miR‐186‐5p), rs3824998 (miR‐221‐3p), rs4768914 (miR‐200c‐3P), rs1046165 (miR‐451a)
72	Case‐control	rs108621 (miR‐193a‐3p, miR‐338‐3p), rs3212986 (miR‐15a)
73	Case‐control	rs3660, rs1044129, rs1053667, rs4901706, rs11337 (miRNA‐binding site polymorphisms)
74	Case‐control	rs1131445 (miR‐135a/135b), rs1051208 (miR‐213), rs743554, rs16870224, rs11515 (miRNA‐binding site polymorphisms)
75	Case‐control	rs1126547 (hsa‐miR‐141, hsa‐miR‐200a), rs2229090 (miR‐1225‐3p, miR‐3123, miR‐3619), rs9914073 (miR‐548c‐3p, miR‐605), rs17339395 (miR‐4299), rs7356 (miR‐3149,miR‐1183), rs1803541 (miR‐568, miR‐802), rs4596 (miR‐518a‐5p, miR‐527, miR‐1205), rs4781563 (miR‐2355‐3p, miR‐4288), rs45522131 (miR‐26a/b, miR‐374a)
76	Case‐control	rs61764370 (let‐7), rs8679 (miR‐145‐3p), rs1804197, rs41116, rs397768, rs4585, rs712, rs16950113 (miRNA‐binding site polymorphisms)
22	Case‐control	rs17281995 (miR‐337, miR‐582, miR‐200a*, miR‐184, miR‐212), rs3135500 (miR‐158, miR‐215, miR‐98, miR‐573), rs1131445 (miR‐135a, miR‐135b, miR‐143, miR‐18, miR‐18a), rs1368439 (miR‐513, miR‐210, miR‐27b, miR‐27a), rs916055 (miR‐588, miR‐183), rs11677 (miR‐187, miR‐638, miR‐154, miR‐453, miR‐296), rs16870224 (miR‐9, miR‐30a‐3p, miR‐30e‐3p), rs1051690 (miR‐618, miR‐612)
77	Case‐control	rs2147578 (miR‐128‐3p,216a‐3p,3681‐3p), rs112462125 (miR‐197‐3p), rs7844527 (miR‐146a‐5p,146b‐5p), rs7814028 (miR‐5001‐3p,miR‐6819‐3p), rs12677572 (miR‐891a‐5p), rs60719452 (miR‐548‐5p,548ab,548ak,548au‐5p,548ay‐5p,548b‐5p,548d‐5p,548i,548y), rs61095617 (miR‐1307‐5p), rs75511849 (miR‐100‐3p)
78	Case‐control	rs88640,3 (miR‐4647, miR‐588, miR‐125, let‐7), rs4077531, rs3733492, rs12732, rs1532602, rs4071, rs17552409, rs17243454, rs4729655, rs7631009, rs6782006, rs974034, rs7372 (miRNA‐binding site polymorphisms)
79	Case‐control	rs712 (miR‐200b, miR‐429, miR‐200c, miR‐193b), rs709805 (miR‐324‐3p), rs2289965 (miR‐142‐3p, miR‐324‐5p), rs3012518 (miR‐299‐3p), rs2839629 (miR‐18a, miR‐18b), rs904960 (miR‐32, miR‐25, miR‐367, miR‐363), rs3734279 (miR‐203), rs354476 (miR‐125a, miR‐125b), rs495714 (miR‐324‐3p, miR‐196b, miR‐196a), rs1048650 (miR‐22), rs496550 (miR‐363), rs473351 (miR‐182)
80	Case‐control	rs2233921 (miR‐3925‐3p, miR‐3140‐3p, miR‐1825, miR‐1825, miR‐3925‐3p, miR‐3140‐3p), rs971 (miR‐4744, miR‐3154, miR‐610, miR‐4744, miR‐3154, hsa‐miR‐610), rs6997097 (miR‐3605‐5p, miR‐3545‐3p, miR‐3605‐5p, miR‐3545‐3p), rs8191670, rs2740439, rs4639, rs1043180, rs1055678, rs1052536 rs2307285, rs2307294, rs1534862, (miRNA‐binding site polymorphisms)
34	Case‐control	rs2279398 miR‐370, rs1047854, rs11206394, rs1128287, rs1131445, rs12462695, rs15049, rs17111100, rs2275085, rs2283606, rs2839531, rs3135499, rs3757417, rs3803098, rs747343, rs9118 (miRNA‐binding site polymorphisms)
81	Case‐control	rs2155209 (miR‐1296, miR‐296‐5p), rs11226 (miR‐296‐5p, miR‐1296), rs1051669 rs11571475, rs7963551, rs12593359, rs7180135, rs45507396, rs8176318, rs13447749, rs9995, rs14448,rs300171, rs300170, rs3218547, rs10131, rs1051685, rs2440, rs1051677, rs897477, rs2035990 (miRNA‐binding site polymorphisms)

a miRNA‐binding site polymorphism: the polymorphism located in miRNA‐binding sites (according to the referenced article).

**Table 2:** 3ʹUTR polymorphisms and colorectal cancer risk (included from first search strategy)

Reference	Study design	rsID
82	Case‐control	rs1058881
83	Case‐control	rs1059234
84	Case‐control	rs731236
85	Case‐control	rs108621
86	Case‐control	rs142559064
40	Case‐control	rs146588909
87	Case‐control	rs17281995
88	Case‐control	rs1801157
89	Case‐control	rs1801157
90	Case‐control	rs1801157
91	Case‐control	rs2075786
44	Case‐control	rs2241703
92	Case‐control	rs3025039
93	Case‐control	rs3025039
94	Case‐control	rs3025039
95	Case‐control	rs3025039
96	Case‐control	rs3212986
50	Case‐control	rs3732360
97	Case‐control	rs3742330
98	Nested case‐cohort	rs5275
99	Case‐control	rs78378222
100	Case‐control	rs5275
101	Case‐control	rs5275
102	Case‐control	rs57898959
103	Case‐control	rs8176318
104	Case‐control	rs696
105	Case‐control	rs713041
106	Case‐control	rs7579
107	Case‐control	rs8878
108	Case‐control	rs9138
109	Case‐control	rs9138
110	Case‐control	CDX2‐G1312T
111	Case‐control	rs868, rs7591
112	Case‐control	rs5275, rs4648298
113	Case‐control	rs67085638, rs77628730
114	Case‐control	rs4648298, rs5276, rs13306035
115	Case‐control	rs1205, rs3093075
116	Case‐control	rs7975232, rs1544410
117	Case‐control	rs16930073, rs8491, rs854551
118	Case‐control	rs11875, rs1042669, rs4149206
119	Case‐control	rs3025040, rs10434, rs3025053
72	Case‐control	rs735482, rs2336219, rs1052133
62	Case‐control	rs12245, rs12587, rs9266, rs1137282
120	Case‐control	rs3742330, rs10719, rs14035, rs11077
121	Case‐control	rs334348, rs334349, rs1590, rs868, rs420549
122	Case‐control	rs11708581, rs12163565, rs390802, rs123598
37	Case‐control	rs2120132, rs2099902, rs10450310, rs10082466
123	Case‐control	rs4846049, rs1537514, rs3737967, rs4846048
124	Case‐control	rs1137188, rs3025039, rs3025040, rs3025053, rs10434
125	Nested case‐cohort	rs11168267, rs11574113, rs731236, rs3847987, rs11574143
66	Case‐control	rs12009, rs700082, rs1057035, rs10404, rs1939861, rs3757261
52	Case‐control	rs7248637, rs11465421, rs10824792, rs2083771, rs1052972
43	Case‐control	rs1707, rs17179101, rs17179108, rs1063320, rs9380142, rs1610696
68	Case‐control	rs4985036, rs9970671, rs11861556, rs17500814, rs12678, rs9129, rs2561819
126	Case‐control	rs2302821, rs45544737, rs34337770, rs7730368, rs16870224, rs4957343, rs9312555
127	Case‐control	rs10849, rs10890324, rs293796, rs7641176, rs293782, rs293783, rs6809452, rs6544991, rs6720549, rs6713506, rs2537742
128	Case‐control	rs2298753, rs706209, rs13420827, rs6058896, rs3827869, rs1832683, rs4846049, rs9282787, rs9332, rs854571, rs1544468, rs10418, rs757158, rs854551, rs3917577

**Table 3:** Genotyping and analysis results of polymorphism with less than four eligible studies

Gene	rsID	Case			Control			References	Sig. in genetic models
		CC	GC	GG	CC	GC	GG		Yes^a^
CD86	rs17281995	7	48	137	0	55	164	87	
		24	161	475	8	114	434	22	
		12	75	217	7	67	181	129	
		CC	TC	TT	CC	TC	TT		
PARP1	rs8679	53	335	687	66	482	873	76	No
		12	60	111	14	86	90	60	
		AA	GA	GG	AA	GA	GG		
VEGF	rs10434	8	57	214	9	83	213	119	No
		19	143	209	11	93	142	124	
		CC	TC	TT	CC	TC	TT		
MLH3	rs108621	219	562	311	300	665	428	85	No
		14	62	124	9	59	132	72	
		CC	CT	TT	CC	CT	TT		
IL‐16	rs1131445	36	110	103	34	159	201	74	No
		65	287	308	53	240	251	22	
		GG	TG	TT	GG	TG	TT		
IL12B	rs1368439	2	29	61	2	35	68	64	No
		21	188	465	15	164	388	22	
		AA	GA	GG	AA	GA	GG		
PTGER4	rs16870224	11	130	523	4	116	439	22	No
		2	68	179	14	109	271	74	
		AA	CA	CC	AA	CA	CC		
*BRCA1*	rs8176318	127	504	484	109	504	560	103	No
		119	445	509	144	634	640	81	
		AA	GA	GG	AA	GA	GG		
VEGF	rs3025053	0	36	243	0	27	278	119	No
		6	91	274	4	67	175	124	
		AA	CA	CC	AA	CA	CC		
MTHFR	rs4846049	79	344	373	83	351	371	123	No
		17	157	276	9	113	278	128	
		AA	AC	CC	AA	AC	CC		Yes^b^
SPP1	rs9138	31	138	99	20	102	152	108	
		20	42	38	19	43	50	109	
		AA	GA	GG	AA	GA	GG		
NOD2	rs3135500	15	37	40	19	48	38	64	Yes^c^
		31	42	15	10	43	35	46	
		120	303	243	81	265	209	22	
		GG	TG	TT	GG	TG	TT		
KRAS	rs61764370	0	66	375	2	35	202	130	No
		1	45	151	2	68	288	56	
		6	167	916	10	215	1200	76	
		AA	AG	GG	AA	AG	GG		
NFKBIA	rs696	55	181	118	155	480	380	104	No
		233	460	308	212	531	262	58	
		57	58	28	22	62	53	57	

VEGF, vascular endothelial growth factor.

a Allelic model, OR: 1.28, 95% CI (1.08‐1.52); Recessive model, OR: 2.23, 95% CI (1.22‐4.07); Dominant model, OR: 1.23, 95% CI (1.01‐1.49); Homozygote, OR: 2.29, 95% CI (1.25‐4.19); Heterozygote CC vs GC OR: 2.06, 95% CI (1.10‐3.83).

b Overdominant model, OR: 1.59, 95% CI (1.19‐2.12).

c AA vs AG OR: 2.50, 95% CI (1.12‐5.57).

**Table 4:** The basic characteristic of included studies (polymorphisms with at least four eligible studies were included)

SNPs	First author	Year	Country	Population subgroup^a^	Case	Study design	Gender	Age	Sample size (case‐control)	Genotyping method	Quality score	References
rs731236	Budhathoki	2016	Japan	East Asian	CRC	Nested case‐control	F/M	40‐69	356/708	TaqMan	8	125
	Takeshige	2015	Japan	East Asian	CRC	Case‐control	F/M	20‐74	685/778	PCR‐RFLP	9	131
	Park	2006	Korea	East Asian	CRC	Case‐control	F/M	23‐81	190/318	PCR‐RFLP	6	132
	Hughes	2011	Czech Republic	European	CRC	Case‐control	F/M	>29	717/615	KASPar	8	133
	Bentley	2012	New Zealand	European	CRC	Case‐control	F/M	—	199/182	TaqMan	7	134
	Gromowski	2016	Poland	European	CRC	Case‐control	—	—	195/390	TaqMan	4	135
	Laczmanska	2014	Poland	European	CRC	Case‐control	F/M	32‐87	157/175	SNaPshot Multiplex Kit	6	84
	Flügge	2007	Russia	European	CRC	Case‐control	F/M	29‐85	256/256	PCR‐RFLP	6	136
	Mahmoudi	2010	Iran	Middle East	CRC	Case‐control	F/M	14‐90	160/180	PCR‐RFLP	6	137
	Moossavi	2017	Iran	Middle East	CRC	Case‐control	F/M	—	100/100	PCR‐RFLP	6	138
	Safaei	2012	Iran	Middle East	CRC	Case‐control	F/M	—	112/112	PCR‐RFLP	6	139
	Atoum	2014	Jordan	Middle East	CRC	Case‐control	F/M	—	93/102	PCR‐RFLP	6	140
	Alkhayal	2016	Saudi Arabia	Middle East	CRC	Case‐control	F/M	21‐89	100/100	Sequencing	5	141
	Gunduz	2012	Turkey	Middle East	CRC	Case‐control	F/M	—	43/42	PCR‐RFLP	6	142
	Yaylım‐Eraltan	2007	Turkey	Middle East	CRC	Case‐control	—	—	26/52	PCR‐RFLP	4	143
	Dilmec	2009	Turkey	Middle East	CRC	Case‐control	F/M	—	56/169	PCR‐RFLP	4	144
	Kupfer	2011	USA	African	CRC	Case‐control	F/M	—	938/811	Sequenom MassARRAY	7	145
	Slattery	2001	USA	Caucasian, African, Hispanic	CRC	Case‐control	F/M	30‐79	427/366	PCR‐RFLP	9	146
	Ochs‐Balcom	2008	USA	Caucasian	CRC	Case‐control	F/M	≥40	250/246	TaqMan	8	147
	Yamaji	2011	Japan	East Asian	Adenoma	Case‐control	F/M	40‐79	684/640	TaqMan	7	148
	Peters	2004	USA	European	Adenoma	Nested Case‐control	F/M	55‐74	716/727	PCR‐RFLP	7	149
	Peters	2004	USA	African	Adenoma	Nested Case‐control	F/M	55‐74	763/774	PCR‐RFLP	7	149
rs30259039	Hofmann	2008	Austria	Caucasian	CRC	Case‐control	F/M	29‐83	427/427	TaqMan	7	150
	Wu	2009	Germany	Caucasian	CRC	Case‐control	F/M	33‐91	157/117	PCR‐RFLP	5	151
	Ungerback	2009	Sweden	Caucasian	CRC	Case‐control	—	—	302/336	MegaBACE™ SNuPe™ Genotyping Kit	5	95
	Bayhan	2014	Turkey	Caucasian	CRC	Case‐control	—	—	43/44	PCR‐RFLP	4	152
	Jannuzzi	2015	Turkey	Caucasian	CRC	Case‐control	F/M	—	103/129	PCR‐RFLP	8	153
	Yang	2017	China	East Asian	CRC	Case‐control	F/M	20‐83	371/246	iMLDR method	7	124
	Bae	2008	Korea	East Asian	CRC	Case‐control	F/M	18‐95	262/229	PCR‐RFLP	5	154
	Chae	2008	Korea	East Asian	CRC	Case‐control	F/M	21‐89	465/413	PCR/DHPLC	4	141
	Jang	2013	Korea	East Asian	CRC	Case‐control	F/M	—	390/492	PCR‐RFLP	6	155
	Lau	2014	Malaysia	South Asian	CRC	Case‐control	—	40‐90	130/212	TaqMan	5	156
	Credidio	2011	Brazil	Caucasian, African	CRC	Case‐control	F/M	25‐97	261/261	PCR‐RFLP	4	157
	Wu	2011	China	East Asian	Adenoma	Case‐control	F/M	18‐75	224/200	TaqMan	8	158
rs3212986	Hou	2014	China	East Asian	CRC	Case‐control	F/M	—	204/204	MALDI‐MS	7	159
	Moreno	2006	Spain	_	CRC	Case‐control	F/M	—	349/300	APEX	7	160
	Ni	2014	China	East Asian	CRC	Case‐control	F/M	—	213/240	TaqMan	8	161
	Yueh	2017	Taiwan	East Asian	CRC	Case‐control	F/M	—	362/362	PCR‐RFLP	7	162
	Zhang	2018	China	East Asian	CRC	Case‐control	F/M	—	200/200	TaqMan	5	72
rs712	Dai	2016	China	Chinese	CRC	Case‐control	F/M	36‐75	430/430	iMLDR	7	62
	Jiang	2015	China	Chinese	CRC	Case‐control	F/M	—	586/476	PCR‐RFLP	5	36
	Landi	2012	Czech Republic	Czechs	CRC	Case‐control	F/M	—	717/1171	KASPar	7	79
	Pan	2014	China	Chinese	CRC	Case‐control	F/M	—	339/313	PCR‐RFLP	7	59
	Schneiderova	2017	Czech Republic	Czechs	CRC	Case‐control	F/M	21‐78	1057/1405	KASPar	6	76
rs5275	Makar (DALS)	2013	USA	Caucasian	CRC	Case‐control	F/M	30‐79	2003/2549	Illumina™ GoldenGate assay	6	163
	Pereira	2010	Portugal	Caucasian	CRC	Case‐control	F/M	50‐75	115/256	PCR‐RFLP	5	100
	Siezen (PPHV)	2006	Netherlands	Caucasian	CRC	Nested Case‐control	F/M	—	200/388	PCR‐RFLP	7	164
	Siezen (DOM)	2006	Netherlands	Caucasian	CRC	Nested Case‐control	F/M	—	442/693	PCR‐RFLP	6	164
	Vogel	2014	Norway	Caucasian	CRC	Case‐control	F/M	50‐64	189/399	KBioscience	8	165
	Zhang	2012	China	East Asian	CRC		F/M	93‐30	343/340		6	101
	Cox	2004	Spain	Caucasian	CRC	Case‐control	F/M	24‐92	290/271	TaqMan	6	166
	Andersen	2013	Denmark	Caucasian	CRC	Case‐Cohort Study	F/M	50‐64	931/1738	KASPar	9	167
	Thompson	2009	USA	Caucasian, African, Other	CRC	Case‐control	F/M	—	421/480	TaqMan	9	168
	Gunter	2006	USA	_	Adenoma	Case‐control	F/M	43‐74	210/197	TaqMan	8	169
	Pereira	2016	Portugal	Caucasian	Adenoma	Case‐control	F/M	50‐75	191/474	—	6	170
	Siezen	2006	Netherlands	Caucasian	Adenoma	Case‐control	F/M	—	378/396	TaqMan	7	171
	Vogel	2014	Norway	Caucasian	Adenoma	Case‐control	F/M	50‐64	983/399	KBioscience	8	165
	Gong	2009	USA	_	Adenoma	Case‐control	F/M	30‐74	162/211	PCR‐RFLP	8	112
	Ali	2005	USA	Caucasian	Adenoma	Nested Case‐control	F/M	55‐74	749/756	TaqMan	7	172
	Ashktorab	2008	USA	African	Adenoma	Case‐control	F/M	—	70/136	TaqMan	7	173
rs4648298	Iglesias	2009	Spain	Caucasian	CRC	Case‐control	F/M	—	284/123	PCR‐RFLP	7	114
	Mosallaei	2018	Iran	Caucasian	CRC	Case‐control	F/M	—	88/88	PCR‐RFLP	5	49
	Ueda	2008	Japan	East Asian	Adenoma	Case‐control	M	47‐59	455/1051	PCR‐RFLP	5	174
	Gong	2009	USA	_	Adenoma	Case‐control	F/M	30‐74	162/211	PCR‐RFLP	8	112
rs1801157	Ramzi	2014	Malaysia	Asian	CRC	Case‐control	F/M	>18	124/173	Illumina's BeadArray	7	175
	Razmkhah	2013	Iran	Caucasian	CRC	Case‐control	—	—	109/262	PCR‐RFLP	4	176
	Amara	2015	Tunis	African	CRC	Case‐control	F/M	—	80/80	PCR‐RFLP	5	177
	Dimberg	2007	Sweden	Caucasian	CRC	Case‐control	F/M	29‐103	258/300	PCR‐RFLP	5	88
	Hidalgo‐Pascual	2007	Spain	Caucasian	CRC	Case‐control	F/M	35‐87	151/141	FRET	4	89
	Shi	2013	Taiwan	Asian	CRC	Case‐control	F/M	>30	349/516	PCR‐DHPLC	6	90

a Different classifications for population subgroup were used for each polymorphism.

**Figure 2:**
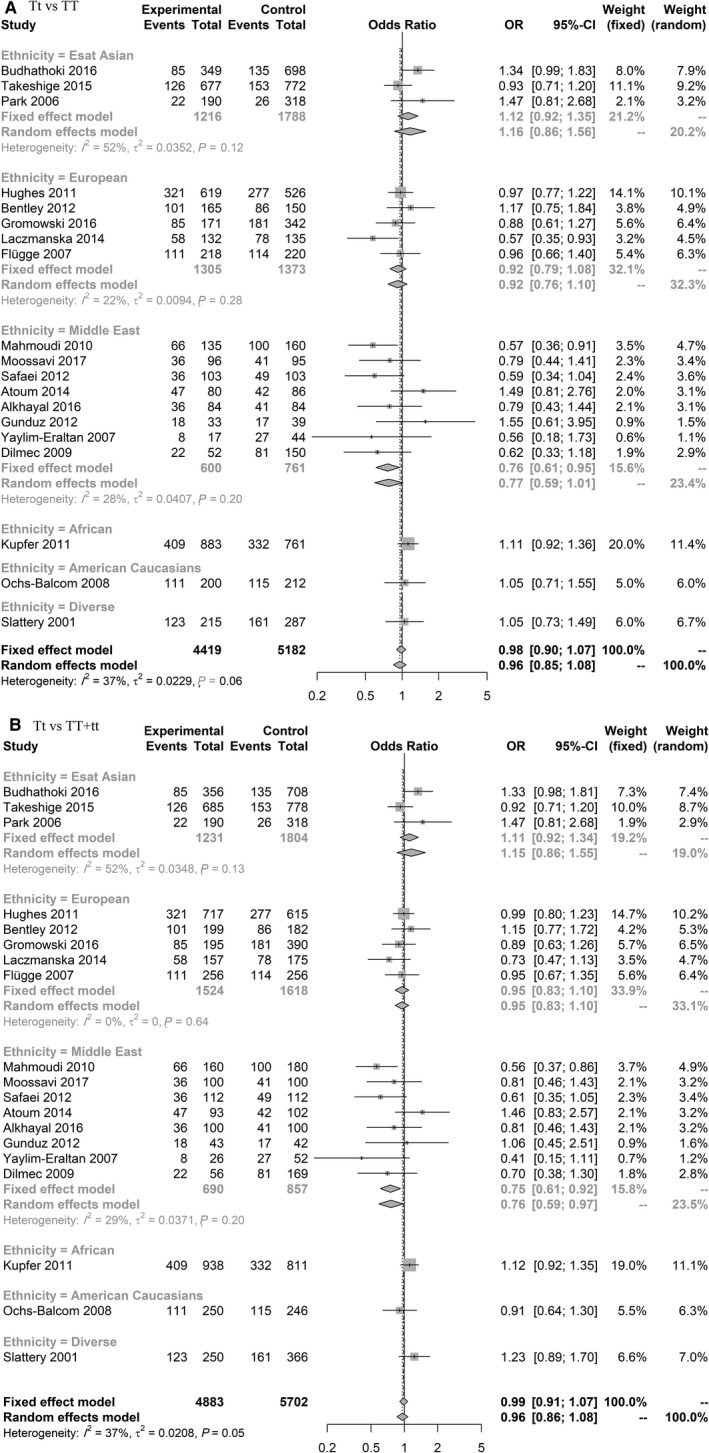
Forest plot related to rs731236 and risk of CRC. A, Heterozygote model. B, Overdominant model

For rs731236 in overall meta‐analysis (based on minor allele; t) no significant result for the risk of CRC was observed, but in subgroup analysis in Middle East population the results were significant in heterozygote (Tt vs TT) (0.76 [0.61‐0.95]) and overdominant models (Tt vs TT + tt) (0.75 [0.61‐0.92]), and borderline significance was observed in dominant model (tt + Tt vs TT) (0.81 [0.66‐1.00]) (Figure 2, Figure S2).

**Figure 3:**
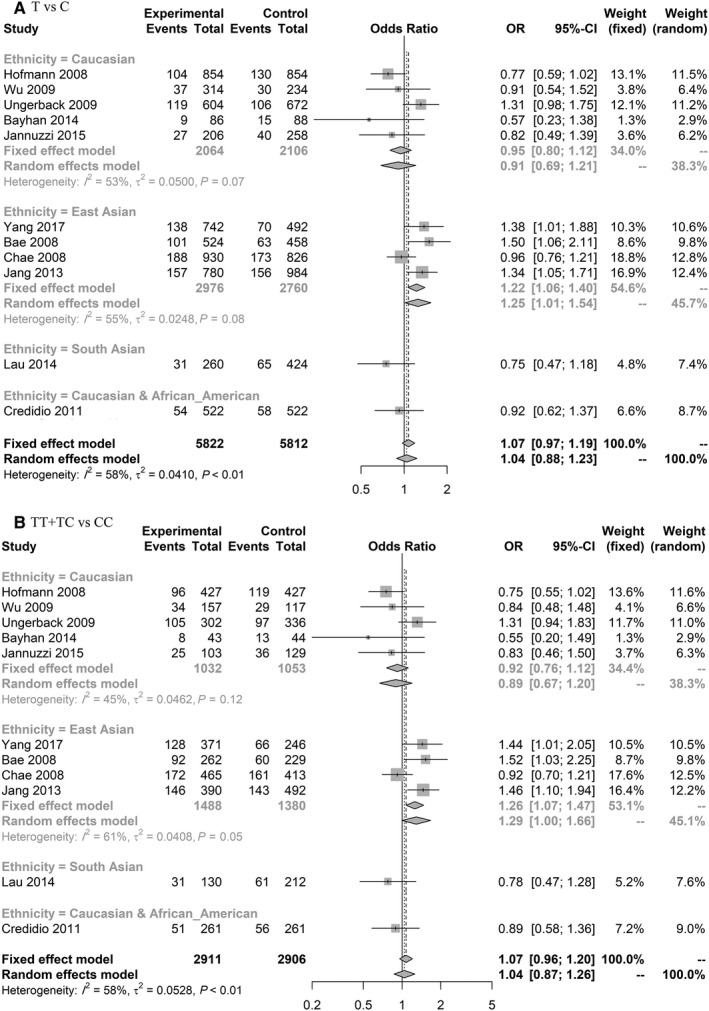
Forest plot related to rs3025039 and risk of CRC. A, Allelic model. B, Dominant model

For rs3025039 in overall, there was no significant association, but subgroup analysis revealed significant results (based on minor allele; T). In East Asian population, the allelic model (T vs C) (1.25 [1.01‐1.54]) significantly increased the risk of CRC and in dominant model (TT + TC vs CC) (1.29 [1.00‐1.66]) there was a trend towards significance (Figure 3, Figure S3).

**Figure 4:**
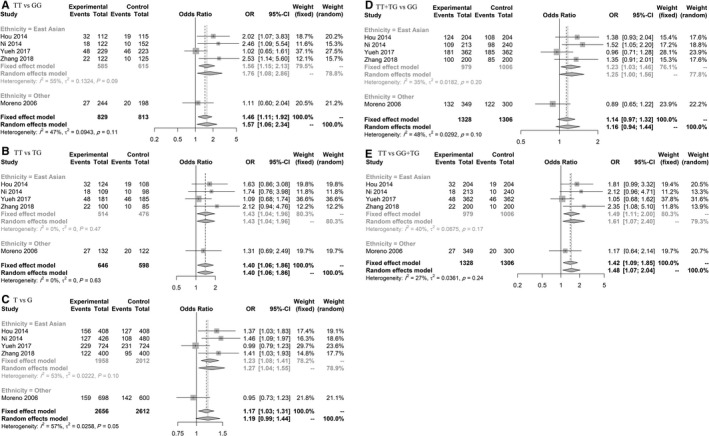
Forest plot related to rs3212986 and risk of CRC. A, Homozygote model. B, TT vs TG model. C, Allelic model. D, Dominant model. E, Recessive model

In meta‐analysis for rs3212986, there were significant results in both overall and subgroup analysis in different genetic models (based on minor allele; T), including homozygote model (TT vs GG) 1.76 (1.08‐2.86) (Figure 4, Figure S4).

**Figure 5:**
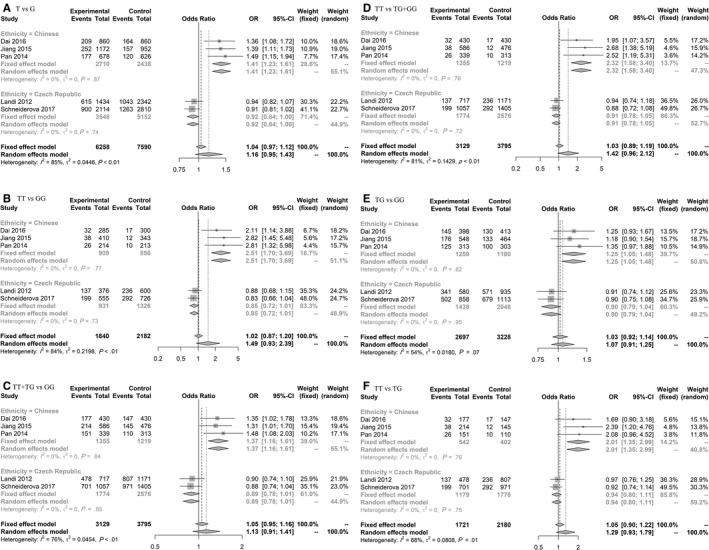
Forest plot related to rs712 and risk of CRC. A, Allelic model. B, Homozygote model. C, Dominant model. D, Recessive model. E, Heterozygote model. F, TT vs TG model

Although we did not find any significant result for rs712 in overall models, subgroup analysis revealed significant and borderline association in Chinese and Czech populations, respectively, on six genetic models (based on minor allele; T), including homozygote model (TT vs GG) in Chinese 2.51 (1.70‐3.69) and in Czech 0.85 (0.72‐1.01) populations (Figure 5, Figure S5).

The allele (A) of rs1801157 polymorphism increased risk of CRC in Asian population, while we did not find any significant results in Caucasian populations (Table 5).

**Table 5:** Meta‐analysis of association between rs1801157 and risk of CRC

Classification	Allelic		Dominant		Recessive		Overdominant	
	OR [95% CI]	Q test *P* value	OR [95% CI]	Q test *P* value	OR [95% CI]	Q test *P* value	OR [95% CI]	Q test *P* value
Caucasian (n = 3)	0.98 [0.82‐1.17]	.89	1.03 [0.83‐1.27]	.90	0.75 [0.44‐1.26]	.45	1.09 [0.88‐1.35]	.76
Asian (n = 2)	**2.28 [1.11‐4.69]**	.02	2.20 [0.66‐7.30]	<.01	**4.94 [1.69‐14.42]**	.58	1.57 [0.28‐8.88]	<.01
Overall (n = 6)	1.56 [0.97‐2.50]	<.01	1.59 [0.93‐2.70]	<.01	2.03 [0.73‐5.63]	<.01	1.24 [0.78‐2.00]	<.01

Classification	Homozygote		AA vs AG		Heterozygote (AG vs GG)	
	OR [95% CI]	Q test *P* value	OR [95% CI]	Q test *P* value	OR [95% CI]	Q test *P* value
Caucasian (n = 3)	0.75 [0.44‐1.29]	.50	0.72 [0.42‐1.25]	.39	1.07 [0.86‐1.33]	.83
Asian (n = 2)	**4.86 [1.63‐14.50]**	.39	**4.96 [1.59‐15.45]**	.90	1.78 [0.38‐8.39]	<.01
Overall (n = 6)	2.31 [0.73‐7.27]	<.01	1.75 [0.69‐4.40]	<.01	1.43 [0.87‐2.35]	<.01

The bold values are statistically significant.

Finally for rs5275 (based on minor allele; C) and rs4648298 (based on minor allele; G), we performed meta‐analysis according to three different subgroup analyses (CRC cases, adenoma, and overall). The results in all different genetic models were not significant except dominant model (0.82 [0.70‐0.97]) in adenoma for rs5275, also the allelic model (C vs T) showed borderline association 0.92 (0.85‐1.00) (Tables 6). For rs4648298 recessive, homozygote, and heterozygote (CG vs GG) models the analysis was not possible, because of zero number in GG genotype in all included studies (Table 7).

**Table 6:** Meta‐analysis of association between rs5275 and risk of CRC (n = 9) and adenoma (n = 7)

Classification	Allelic		Dominant		Recessive		Overdominant	
	OR [95% CI]	Q test *P* value	OR [95% CI]	Q test *P* value	OR [95% CI]	Q test *P* value	OR [95% CI]	Q test *P* value
CRC	1.03 [0.98‐1.09]	.16	1.03 [0.92‐1.16]	.18	1.04 [0.97‐1.12]	.38	0.97 [0.90‐1.04]	.70
Adenoma	0.92 [0.85‐1.00]	.78	**0.82 [0.70‐0.97]**	.19	0.94 [0.83‐1.05]	.07	0.90 [0.71‐1.15]	<.01
Overall	1.00 [0.95‐1.04]	.16	0.96 [0.87‐1.05]	.05	1.01 [0.95‐1.08]	.09	0.95 [0.86‐1.04]	.01

Classification	Homozygote		CC vs CT		Heterozygote (CT vs TT)	
	OR [95% CI]	Q test *P* value	OR [95% CI]	Q test *P* value	OR [95% CI]	Q test *P* value
CRC	1.05 [0.93‐1.18]	.13	1.04 [0.96‐1.13]	.59	1.01 [0.90‐1.15]	33
Adenoma	0.85 [0.71‐1.02]	.38	1.06 [0.83‐1.36]	<.01	0.79 [0.59‐1.06]	<.01
Overall	0.98 [0.89‐1.09]	.10	1.03 [0.93‐1.14]	.03	0.88 [0.76‐1.03]	.02

The bold values are statistically significant.

**Table 7:** Meta‐analysis of association between rs4648298 and risk of CRC (n = 2) and adenoma (n = 2)

Classification	Allelic		Dominant/Overdominant/Heterozygote^a^	
	OR [95% CI]	Q test *P* value	OR [95% CI]	Q test *P* value
CRC	1.93 [0.21‐17.52]	<.01	0.47 [0.04‐5.39]	<.01
Adenoma	1.02 [0.48‐2.18]	.99	0.98 [0.46‐2.11]	.99
Overall	1.41 [0.49‐4.05]	<.01	1.47 [0.47‐4.63]	<.01

a These models had similar results, because of zero number in GG genotype.

## 4 DISCUSSION

This study aimed to investigate miRNA‐binding site polymorphisms and risk of CRC, which may potentially play roles in various conditions. The effects shown for these polymorphisms associated with miRNA:mRNA interactions. Polymorphisms in miRNA‐binding site can negatively or positively influence these interactions by different mechanisms such as effect of hybrid stability, target sites accessibility, local RNA secondary structure, and structural accessibility. Among 222 included polymorphisms, 25 were eligible for inclusion in our secondary search strategy. Fourteen polymorphisms, with less than four eligible studies, were included in the pooled analysis. The rs17281995 polymorphism is located in 3'UTR of CD86 gene and binding site of miR‐337 and miR‐582.^22^ The minor allele (C) of rs17281995 polymorphism increased the risk of CRC in different genetic models. Although the results are based on limited number of studies but the strong association is noteworthy. This was also observed in the previous review based on two included articles.^129^ The nonsignificant results are not conclusive and cannot rule out the association between these polymorphisms and the risk of CRC, because of limited number of included studies and also ethnic differences in studied populations. Further studies need to confirm these results. In addition, seven polymorphisms, with more than four eligible studies, were included in the final meta‐analysis.

The rs731236 polymorphism is located in 3'UTR of vitamin D receptor gene. Its downregulation is related to cancer progression.^178^ There are several previous meta‐analyses on the role of rs731236 on CRC risk. Most of the previous meta‐analyses^179^, ^180^, ^181^, ^182^, ^183^ found no significant association between the risk of CRC and rs731236. While Serrano et al in their meta‐analysis^184^ found significant results based on analyzing both of colorectal cancer and adenoma. Therefore, all previous meta‐analysis results were according to fewer included studies, the overall CRC population and no subgroup analysis were carried out and in some studies adenoma was also included for calculating the risk of CRC. In our study, we carried out subgroup analysis based on different ethnicity and found that the results were different after stratification according to ethnicity. While in overall analysis our results are in line with the previous meta‐analysis, showing no relation between the risk of CRC and rs731236 polymorphism. In Middle East population we observed a significant association between this polymorphism and CRC. This result was not reported previously. We also found a heterozygote advantage for the risk of CRC with heterozygote (Tt) showing protective effects compared with homozygotes (TT, tt). Similarly, in a study on pediatric solid tumor, the heterozygote model decreased the risk of CRC compared to homozygote model. The survival rate of subjects with CRC was significantly decreased in heterozygote model compared to homozygote model.^185^ More studies are needed to specify the reason for our interesting observation.

In overall analysis, based on 11 included studies, rs3025039 was not related to the risk of CRC, but is showing association in Caucasian and East Asian populations. Based on subgroup analysis, minor allele in East Asian was related to an increased risk of CRC. This SNP is located in 3'UTR of vascular endothelial growth factor gene which may affect hsa‐miR‐591 target sites.^186^ This gene affects angiogenesis, tumor growth, and metastasis.^187^ It is also related to CRC outcomes and treatment.^124^ Thus the association between rs3025039 and CRC risk may be related to the effect of this SNP on miRNA:mRNA interactions. However, in the previous meta‐analysis with five included studies, no significant association was found between this polymorphism and risk of CRC.^188^ This might be due to heterogeneity of their data in different populations requiring further subgroup analysis.

According to the results based on five included studies, rs3212986 increased the risk of CRC in all genetic models, which was similar to previous meta‐analysis,^189^ we also found to the same results in East Asian population. This polymorphism is located in binding site of miR‐15a in 3'UTR of ERCC1.^72^ The polymorphisms and mRNA level of this gene had previously been investigated in CRC.^190^

For rs1801157 minor allele (A) increased risk of CRC was observed in Asian population. This result is similar to previous meta‐analysis by Xu,^191^ which found significant association in non‐Caucasian populations. This polymorphism is located in 3'UTR of CXCL12 in a putative miRNA‐binding site for miR‐941.^192^ The effect of CXCL12 polymorphisms on CRC was previously observed in different studies. The CXCL12 binds to CXCR4 and affects different clinical features of cancers such as progression, angiogenesis, and metastasis.^193^ Thus the observed association for rs1801157 A allele and CRC may be related to its effect on miRNA:mRNA interactions and CXCL12 expression.

We also found no significant association between rs712 and risk of CRC, in the overall meta‐analysis of five included studies. However, subgroup analysis revealed remarkable and completely different results in Chinese and Czech Republic populations. In Chinese, we observed a strong risk while in Czech population a protective effect was shown in all various models. There is one study similar to our results which confirm the increase risk of this polymorphism in Chinese population.^194^ In two other meta‐analyses it has been reported that this polymorphism may increases the overall risk of different types of cancers in the Chinese population.^195^, ^196^ This variant is within let‐7 KRAS binding site. KRAS, is an important oncogene, which has been previously described to be associated with different types of cancers. This gene influence cancer cells differentiation and proliferation, and is highly mutated in many type of cancers such as CRC.^197^, ^198^ Based on our results differences between populations should be considered for the effect of this binding site polymorphism in future studies.

In addition, our results (based on 10 eligible studies) showed that rs5275 was not related to the risk of CRC. While the minor allele of rs5275 may have a protective effect on the risk of adenoma. This polymorphism is located in COX‐2 gene at miR‐542‐3p target site. COX‐2 is usually overexpressed in colorectal adenoma patients,^199^ and has effect on pro‐inflammatory prostaglandins and links between inflammation and cancer progression.^200^ Therefore, the minor allele of rs5275 may be associated with a decreased risk of colorectal adenoma by downregulating COX‐2 expression.

### 4.1 Strength and limitations

Our study had several advantages: First, this is the first systematic review for evaluating the role of miRNA‐binding site polymorphisms on CRC susceptibility, and 25 polymorphisms were included in our pooled analysis. Second, to reduce the publication biases and include all relevant documents we carried out a systematic search on four common databases, as well as other sources such as references of relevant reviews. Third, there was no language bias, we included all relevant documents without any language restriction. Fourth, our study has high power and strength reliability because of our comprehensive and double search strategies and subgroup analyzing based on different ethnicity. Fifth, to reduce binding site false positive prediction, related to bioinformatics tools, we only included polymorphisms located in miRNA‐binding site or 3'UTR (stated at least in two of the included documents).

There are also some limitations in our study. First, based on insufficient data, it was mandatory to exclude some relevant documents. Second, some polymorphisms had two or three included article. Third, CRC is a multifactorial disease and we only included genetic effect.

## 5 CONCLUSION

miRNA‐binding site polymorphisms in this meta‐analysis showed significant association with CRC in different populations. Interestingly, rs731236 polymorphism showed a significant association with CRC in Middle East population with a heterozygote advantage. The minor allele in the East Asian populations for rs3025039, rs3212986, and rs712, and also in Asian population for rs1801157, increased the risk of CRC. The minor allele of rs712 may have a protective effect on the risk of CRC in Czech populations, while rs17281995 showed risk effect in the European population. Finally, it can be concluded that these miRNA‐binding site polymorphisms play different roles on the risk of CRC in various populations which should be considered in data analysis and interpretation in the future studies.

## CONFLICT OF INTEREST

The authors declare that there is no conflict of interest.

## Data Availability

Data sharing is not applicable to this article as no new data were created or analyzed in this study.
