# Supplementary material for: MicroRNA‐binding site polymorphisms and risk of colorectal cancer: A systematic review and meta‐analysis
Source: Cancer Med. 2019 Oct 21;8(17):7477–99. doi: 10.1002/cam4.2600 (PMC6885874; doi:10.1002/cam4.2600)
Supplement: Supplementary file 1 [file CAM4-8-7477-s001.docx]

A.


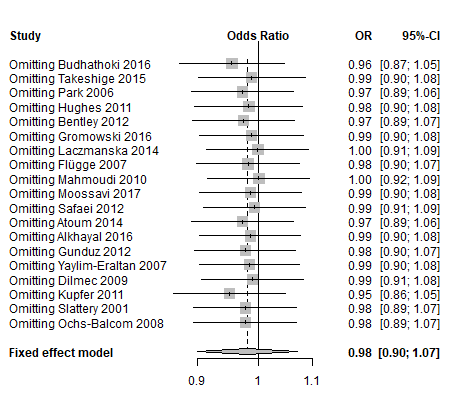


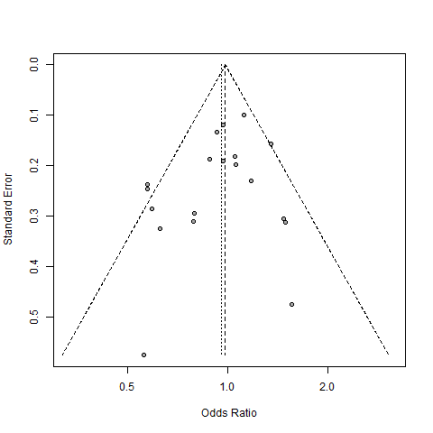


B.


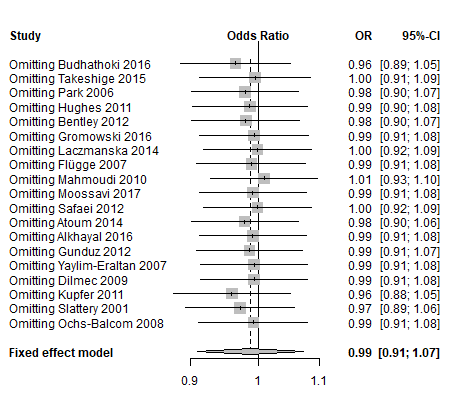


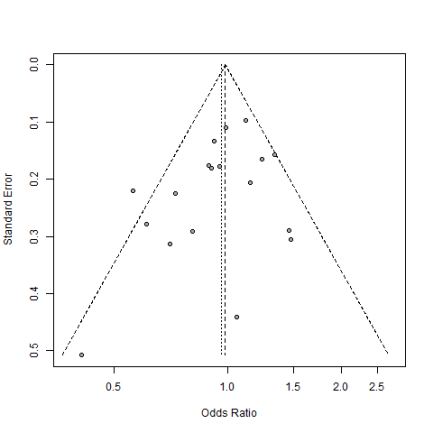


Supporting Information Figure S2. Forest and funnel plots related to rs731236 and risk of CRC. A. heterozygote model B. Over-dominant model
