# Supplementary material for: MicroRNA‐binding site polymorphisms and risk of colorectal cancer: A systematic review and meta‐analysis
Source: Cancer Med. 2019 Oct 21;8(17):7477–99. doi: 10.1002/cam4.2600 (PMC6885874; doi:10.1002/cam4.2600)
Supplement: Supplementary file 2 [file CAM4-8-7477-s002.docx]

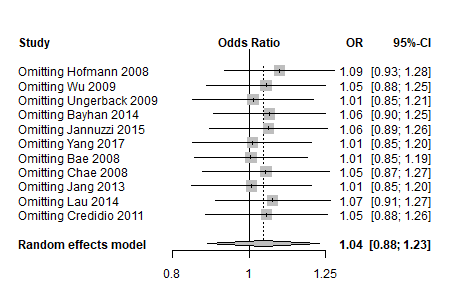


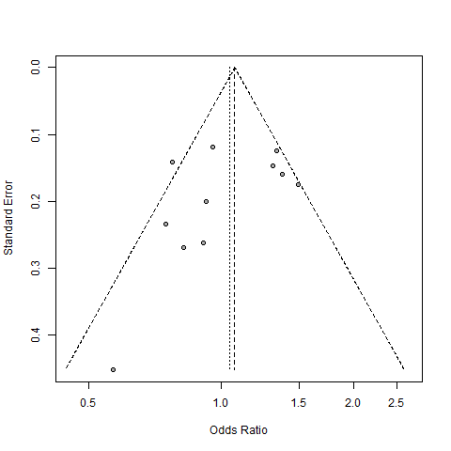


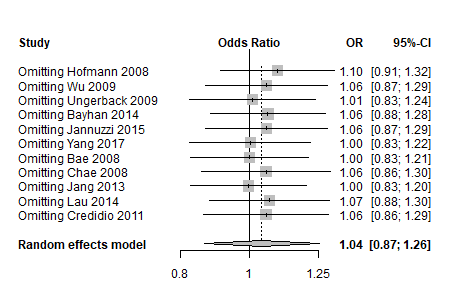


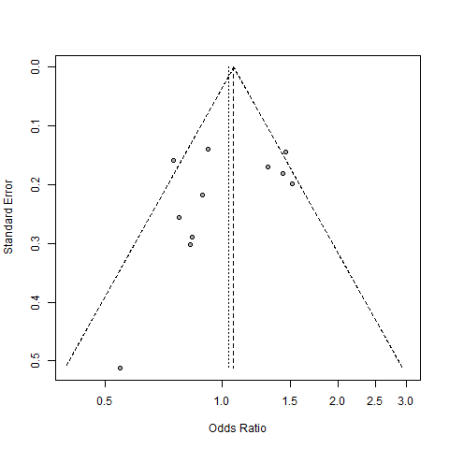


Supporting Information Figure S3. Forest and funnel plots related to rs30259039 and risk of CRC. A. Allelic model B. Dominant model.
