# Supplementary material for: MicroRNA‐binding site polymorphisms and risk of colorectal cancer: A systematic review and meta‐analysis
Source: Cancer Med. 2019 Oct 21;8(17):7477–99. doi: 10.1002/cam4.2600 (PMC6885874; doi:10.1002/cam4.2600)
Supplement: Supplementary file 3 [file CAM4-8-7477-s003.docx]

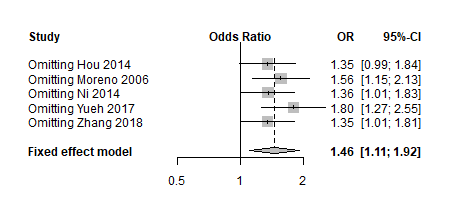


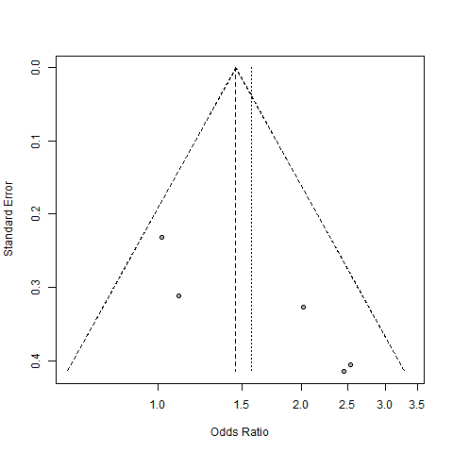


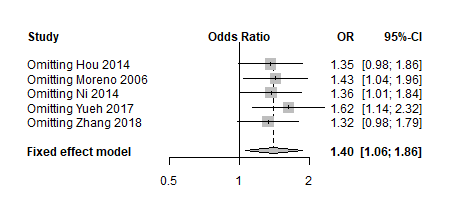


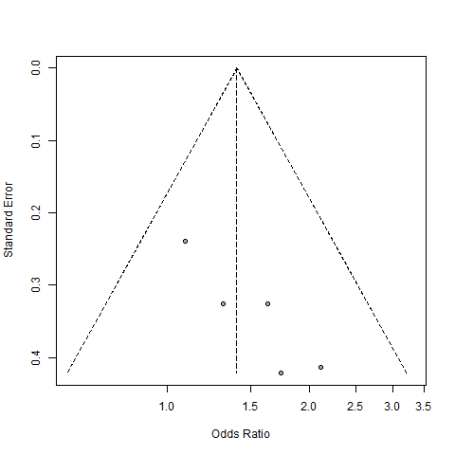


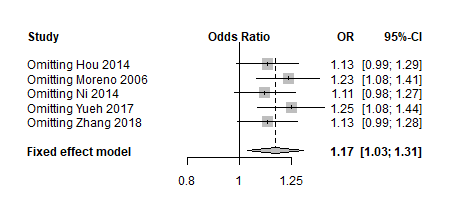


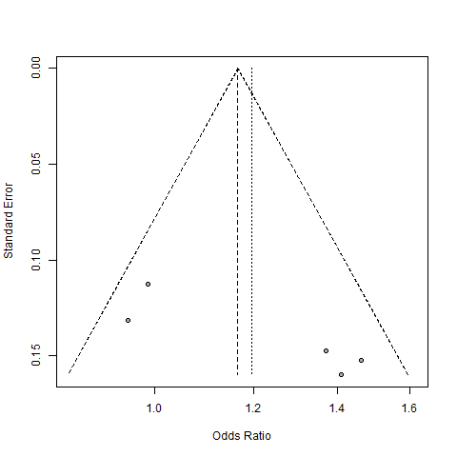


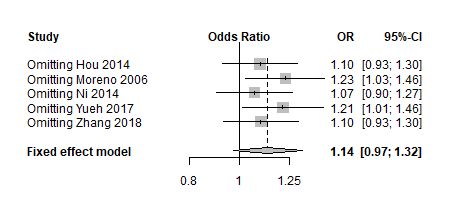


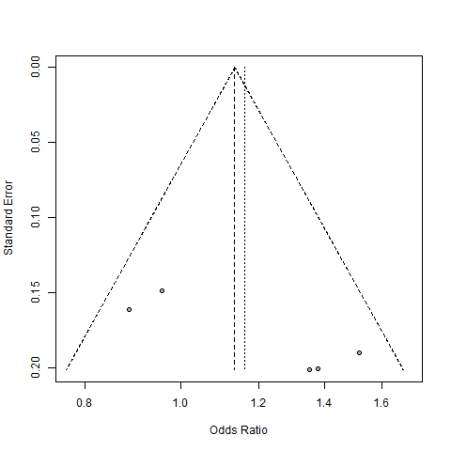


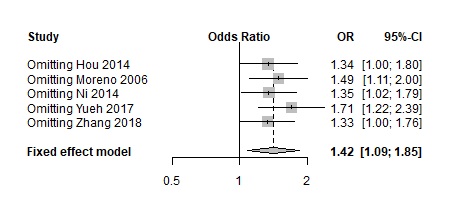


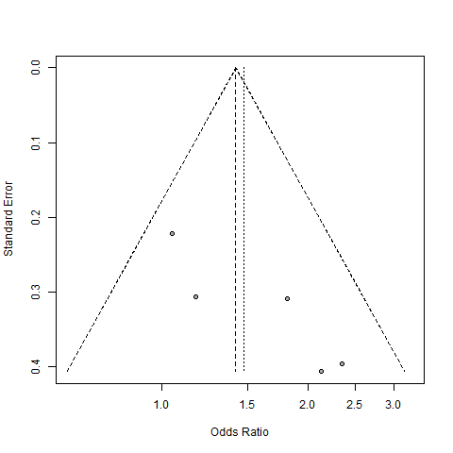


Supporting Information Figure S4. Forest and funnel plots related to rs3212986 and risk of CRC. A. Homozygote model B. TT vs. TG model C. Allelic model D. Dominant model E. Recessive model
