# Supplementary material for: MicroRNA‐binding site polymorphisms and risk of colorectal cancer: A systematic review and meta‐analysis
Source: Cancer Med. 2019 Oct 21;8(17):7477–99. doi: 10.1002/cam4.2600 (PMC6885874; doi:10.1002/cam4.2600)
Supplement: Supplementary file 4 [file CAM4-8-7477-s004.docx]

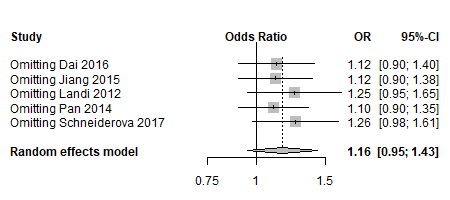


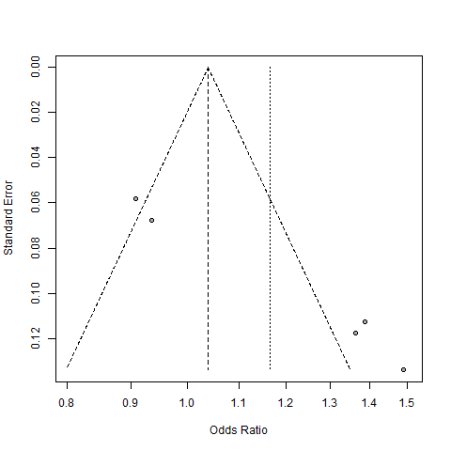


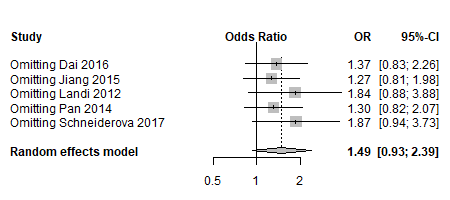


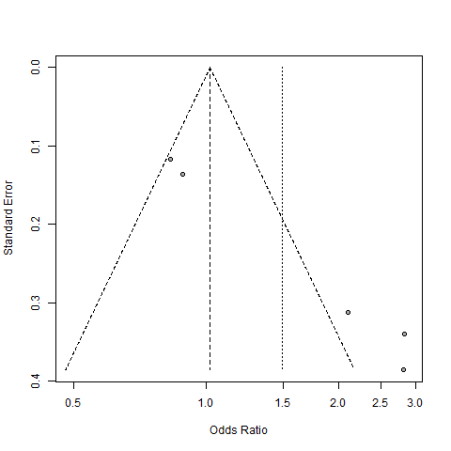


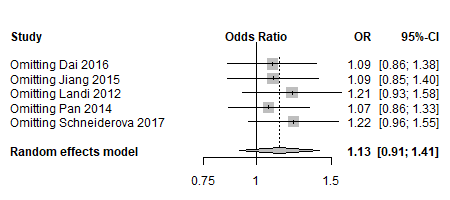


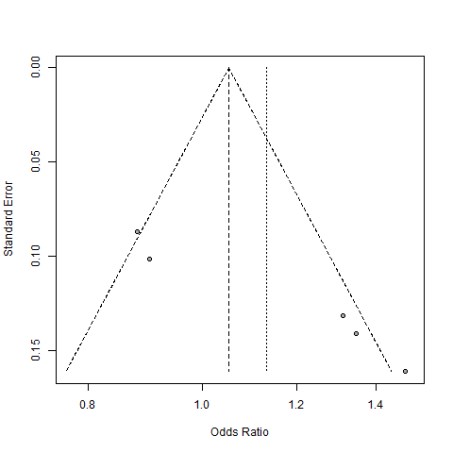


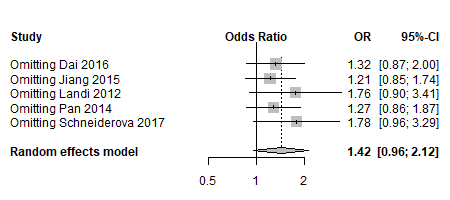


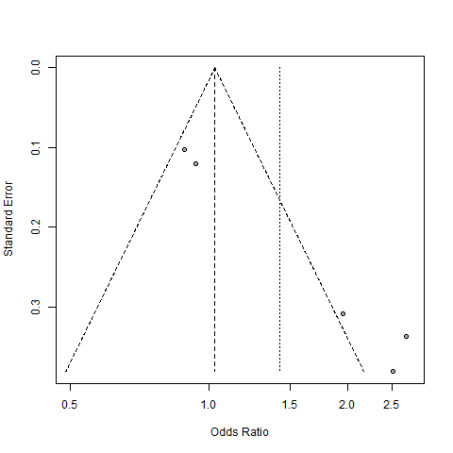


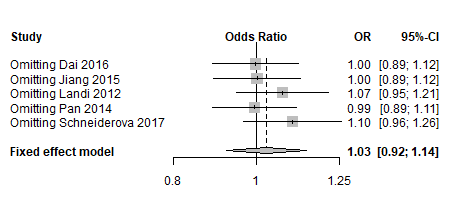


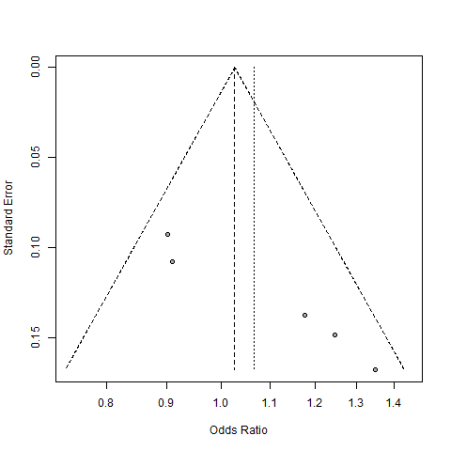


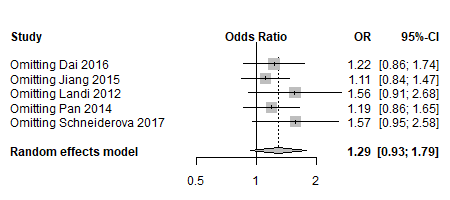


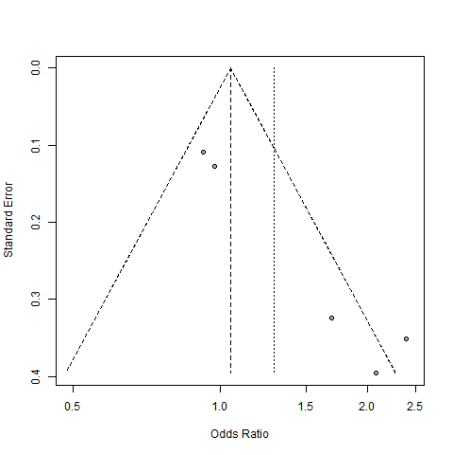


Supporting Information Figure S5. Forest and funnel plots related to rs712 and risk of CRC. A. Allelic model B. Homozygote model C. Dominant model D. Recessive model E and F. TT vs. TG model
