# Supplementary material for: MicroRNA‐binding site polymorphisms and risk of colorectal cancer: A systematic review and meta‐analysis
Source: Cancer Med. 2019 Oct 21;8(17):7477–99. doi: 10.1002/cam4.2600 (PMC6885874; doi:10.1002/cam4.2600)
Supplement: Supplementary file 5 [file CAM4-8-7477-s005.docx]

Supporting Information Table S1. Search strategy used for PubMed database

| Search items | Number |
| --- | --- |
| “Colorectal Neoplasm”[mesh] OR CRC[tiab] OR “colorectal disease”[tiab] | 1 |
| "Rectal Neoplasms"[Mesh] OR “Cancer of the Rectum” | 2 |
| "Sigmoid Neoplasms"[Mesh] OR “Cancer of Sigmoid” OR “Cancer of the Sigmoid” | 3 |
| "Colonic Neoplasms"[Mesh] OR “carcinoma coli”[tiab] OR “Cancer of Colon” OR “Cancer of the Colon” | 4 |
| “large intestine tumor”[tiab] | 5 |
| “Hereditary Nonpolyposis Colorectal Neoplasms”[tiab] OR HNPCC[tiab] | 6 |
| FAP[tiab] OR (polypos*[tiab] AND familial[tiab]) OR (Polyposis[tiab] AND “Adenomatous Intestinal”[tiab]) | 7 |
| (Syndrome[tiab] AND Lynch[tiab]) | 8 |
| (Polypos*[tiab] AND Myh-Associated[tiab]) | 9 |
| 1 OR 2 OR 3 OR 4 OR 5 OR 6 OR 7 OR 8 OR 9 | 9 |
| bowel*[tiab] OR intestin*[tiab] colorect*[tiab] OR rectum[tiab] OR rectal[tiab] OR colon*[tiab] OR col*[tiab] OR anal[tiab] OR anus[tiab] OR retrorectal[tiab] OR abdom*[tiab] OR sigmoid*[tiab] | 10 |
| cancer*[tiab] OR neoplas*[tiab] OR tumor*[tiab] OR tumour*[tiab] OR mass[tiab] OR carcinom*[tiab] OR sarcom*[tiab] OR adenocarcinom*[tiab] OR adenomatosum[tiab] OR adenom*[tiab] OR lesion*[tiab] OR malignan*[tiab] OR polyp[tiab] OR polyps[tiab] OR polypo*[tiab] OR ONCOL*[tiab] OR carcinogenesis[tiab] OR “hereditary non polyp*”[tiab] OR “hereditary nonpolyp*”[tiab] OR “hereditary Polyposis” [tiab] OR “adenomatous polyp*”[tiab] OR papilloma[tiab] OR pseudopolyp[tiab] | 11 |
| 10 AND 11 | 12 |
| 9 OR 12 | 13 |
| "MicroRNAs"[Mesh] OR “micro RNA”[tiab] OR (RNA[tiab] OR RNAs[tiab] AND Micro[tiab]) OR miRNA[tiab] OR miRNAs[tiab] | 14 |
| stRNA[tiab] OR (RNA[tiab] AND “Small Temporal”[tiab]) OR (“Temporal RNA”[tiab] AND Small[tiab]) OR “Small Temporal RNA”[tiab] | 15 |
| miR[tiab] OR miRs[tiab] OR MIRN[tiab] OR MIRNS[tiab] | 16 |
| “hsa miR”[tiab] OR hsa-miR[tiab] | 17 |
| let[tiab] OR lin[tiab] | 18 |
| 3'UTR[tiab] OR (“Untranslated Region”[tiab] AND 3'[tiab]) OR (UTR[tiab] AND 3'[tiab]) OR (UTRs[tiab] AND 3'[tiab]) | 19 |
| “Coding sequence”[ti] OR “coding region”[ti] OR CDS[ti] OR “coding DNA sequence”[ti] OR Exon[ti] | 20 |
| “binding site*”[tiab] OR “target site*”[tiab] OR “combining site*”[tiab] | 21 |
| 14 OR 15 OR 16 OR 17 OR 18 OR 19 OR 20 OR 21 | 22 |
| "Polymorphism, Single Nucleotide"[Mesh] OR (Polymorphism*[tiab] AND Single[tiab] AND Nucleotide[tiab]) ] | 22 |
| SNP[tiab] OR SNPs[tiab] | 23 |
| rs[tiab] | 24 |
| genotyp*[tiab] | 25 |
| Polymorphism*[tiab] | 26 |
| Variation*[tiab] | 27 |
| Variant*[tiab] | 28 |
| ((genes[tiab] OR gene[tiab] OR genetic*[tiab] OR DNA[tiab] OR Nucleotide[tiab]) AND (mutation*[tiab] OR diversit*[tiab] OR polymorphism*[tiab])) | 29 |
| 22 OR 23 OR 24 OR 25 OR 26 OR 27 OR 28 OR 29 | 30 |
| 13 AND 20 AND 30 | 31 |
